# Supplementary material for: Antiviral Activity of Zinc Oxide Nanoparticles against SARS-CoV-2
Source: Int J Mol Sci. 2023 May 8;24(9):8425. doi: 10.3390/ijms24098425 (PMC10179150; doi:10.3390/ijms24098425)
Supplement: Supplementary file 1 [file ijms-24-08425-s001.zip › ijms-2208111-supplementary.pdf]

Supplementary files

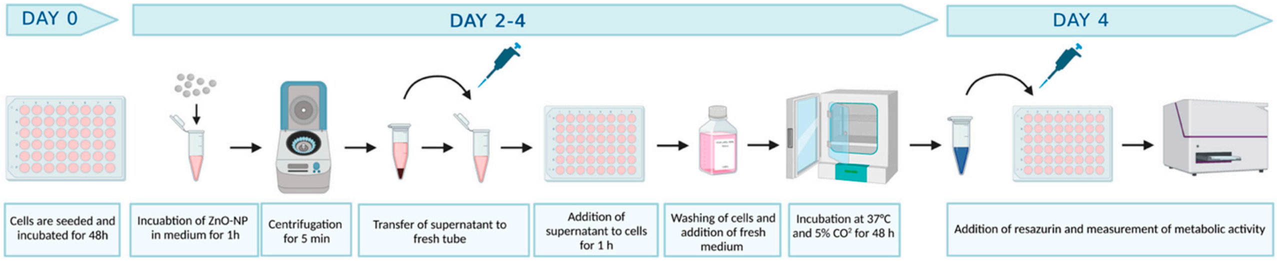

Figure S1. Schematic workflow of the metabolic interference assay. Created with Biorender.com

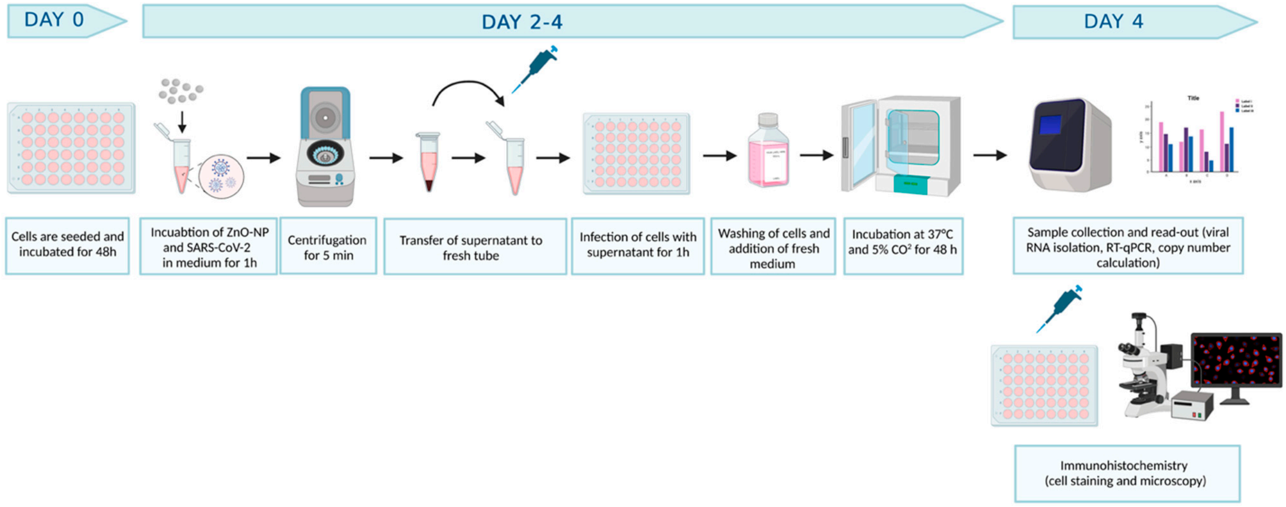

Figure S2. Schematic workflow of the virus neutralization assay using Calu-3 cells infected with ZnO-NP pre-treated SARS-CoV-2. Created with Biorender.com

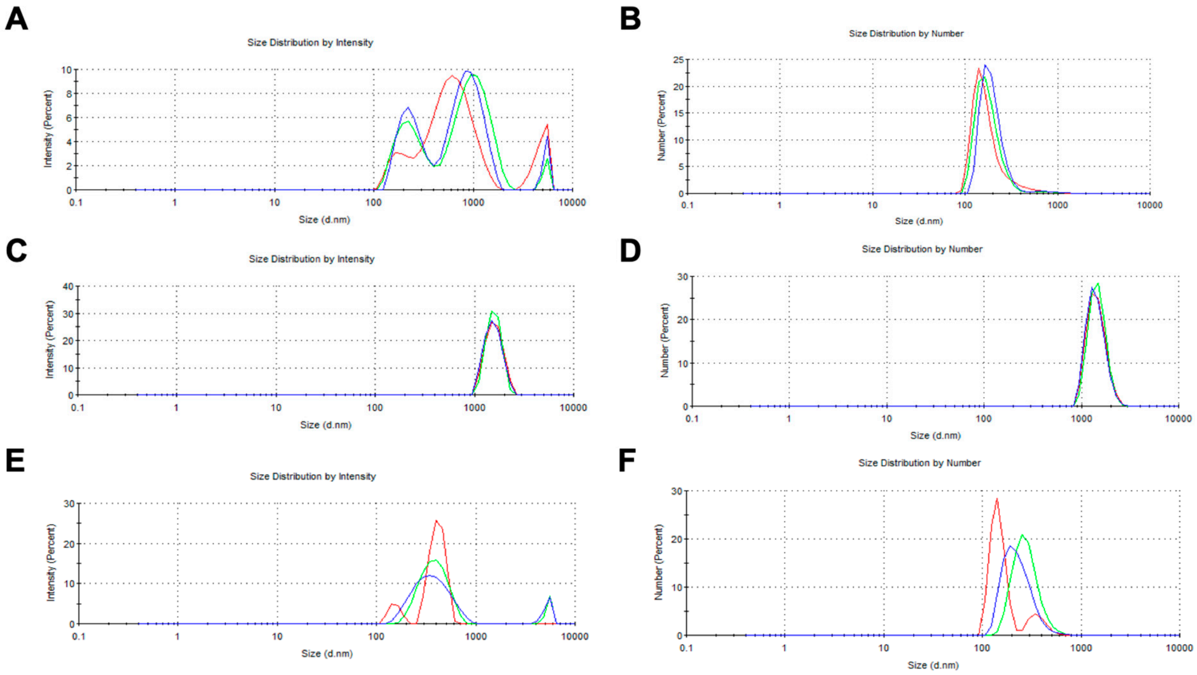

Figure S3. Dynamic Light Scattering (DLS) size distribution from the tested ZnO-NPs. A, C, E showing the DLS distribution by intensity (n=3). B, D, F showing the DLS distribution by number (n=3). ZnO-NP-45 (A, B). ZnO-NP-76 (C, D). ZnO-NP-ref (E, F).

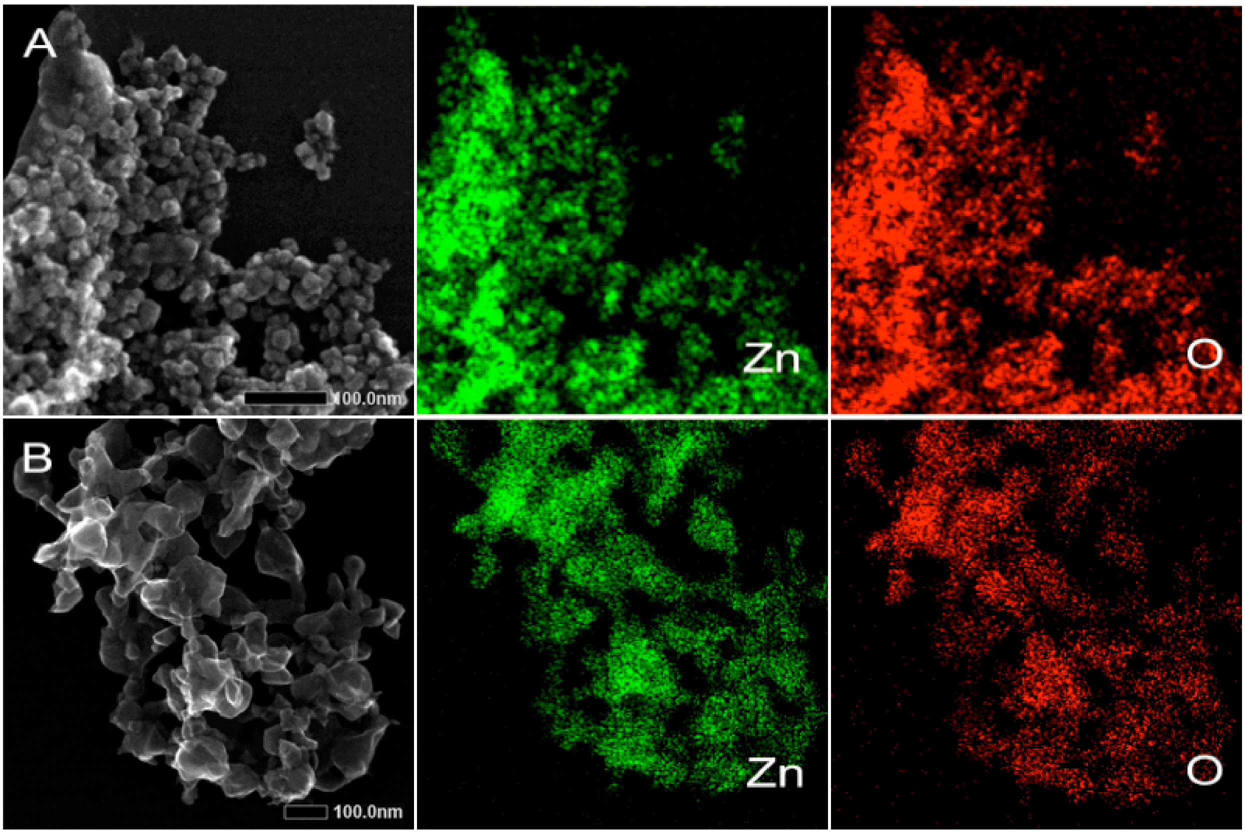

Figure S4. STEM images of the different ZnO-NP with the corresponding EDX intensity maps. The presence of Zn and O is shown for all NP tested. A: ZnO-NP-45, B: ZnO-NP-76

Table S1 Virus copy numbers at 48 h post infection of cell culture with the SARS-CoV-2 Delta containing supernatant after pre-treatment with ZnO-NPs for each replicate (according to Figure 4 b and d in main text).

ZnO-NP-45			ZnO-NP-76			pos Ctrl	
20 mg/ml	10 mg/ml	5 mg/ml	20 mg/ml	10 mg/ml	5 mg/ml		
5.27E+01	2.80E+01	4.11E+08	1.73E+03	2.10E+08	1.07E+08	9.85E+07	
2.18E+01	3.28E+01	2.13E+08	3.70E+07	6.64E+07	1.62E+08	6.47E+07	
2.64E+01	2.44E+01	6.22E+01	4.75E+01	4.41E+01	5.60E+07	4.91E+07	
2.59E+01	4.27E+01	5.97E+01	4.53E+01	4.78E+01	1.41E+08	6.30E+07	
6.59E+01	2.59E+01	8.58E+07	2.88E+01	5.86E+07	5.63E+07	1.07E+08	
9.10E+00	1.33E+01	9.47E+01	6.34E+01	3.14E+01	4.75E+01	7.05E+08	
3.37E+01	3.42E+01	1.15E+09	4.36E+01	1.09E+02	4.03E+08	6.51E+08	
2.30E+01	2.54E+08	5.49E+02	1.22E+00	7.00E+08	4.90E+02	5.98E+08	
1.16E+01	6.77E+01	7.47E+01	8.04E+08	1.40E+09	2.97E+01	3.95E+08	
2.86E+01	1.48E+02	4.22E+06	9.41E+02	6.38E+08	7.83E+08	4.87E+08	
1.18E+01	4.03E+00	2.64E+01	5.04E+00	1.20E+01	1.75E+01	1.04E+08	
1.19E+01	7.77E+00	2.66E+01	7.37E+00	1.05E+01	2.27E+01	3.70E+07	
1.47E+01	8.92E+00	2.66E+01	6.22E+00	1.79E+01	2.88E+01	6.09E+02	
4.47E+00	9.41E+00	2.13E+01	5.48E+00	1.28E+01	2.49E+01	3.44E+07	
1.22E+01	9.85E+00	2.92E+01	6.90E+00	1.19E+01	2.03E+01	4.00E+07	
2.36E+01	1.69E+07	1.25E+08	5.60E+07	2.05E+08	1.14E+08	2.29E+08	**Mean**

Table S2 Virus Input used for cell infection after pre-treatment with ZnO-NPs for 1 h and subsequent centrifugation for each experimental series (according to Figure 4 a and c in main text).

VI ZnO-NP-45			VI ZnO-NP-76			VI pos Ctrl
20 mg/ml	10 mg/ml	5 mg/ml	20 mg/ml	10 mg/ml	5 mg/ml	
6.77E+02	8.35E+02	1.39E+03	5.52E+02	1.80E+03	3.20E+04	3.20E+04
8.69E+02	2.36E+03	4.65E+03	1.67E+03	4.65E+03	1.75E+04	6.34E+05
3.22E+02	1.00E+03	3.05E+03	8.30E+02	2.36E+03	4.05E+03	6.68E+05

Table S3 Virus copy numbers at 48 h post infection with SARS-CoV-2 Delta suspension after pre-treatment with ZnO-NPs for each replicate (according to Figure 4 f in main text).

ZnO-NP-ref				
20 mg/ml	10 mg/ml	5 mg/ml	pos Ctrl	
1.83E+04	3.90E+01	2.20E+05	8.14E+06	
3.85E+01	2.20E+01	8.99E+04	1.48E+07	
9.35E+01	9.92E+01	not detectable	1.06E+07	
3.12E+05	2.39E+05	2.75E+05	6.42E+06	
2.30E+04	5.31E+04	8.19E+05	1.32E+06	
7.06E+04	5.85E+04	3.51E+05	8.26E+06	**Mean**

Table S4 Virus Input used for cell infection after pre-treatment with ZnO-NPs for 1 h and subsequent centrifugation (according to Figure 4 e in main text).

VI ZnO-NP-ref			VI pos Ctrl
20 mg/ml	10 mg/ml	5 mg/ml	
4.11E+02	4.39E+02	2.24E+03	7.93E+04

Table S5 Virus copy numbers after pre-treatment with ZnO-NPs at three different concentrations at 48 h post infection of cell culture with the SARS-CoV-2 Omicron-containing supernatant for each replicate (according to Figure 5 b and d in main text).

ZnO-NP-45			ZnO-NP-76			pos Ctrl	
20 mg/ml	10 mg/ml	5 mg/ml	20 mg/ml	10 mg/ml	5 mg/ml		
4.31E+02	6.35E+02	1.37E+08	1.03E+04	1.46E+02	5.50E+07	8.89E+07	
3.90E+02	1.67E+02	7.69E+02	1.87E+02	1.25E+02	6.52E+02	1.91E+08	
4.69E+02	5.05E+02	1.14E+07	6.57E+06	2.04E+02	4.45E+07	7.25E+07	
3.04E+02	2.77E+02	5.83E+05	2.90E+02	1.18E+02	2.58E+07	1.92E+08	
1.28E+02	3.14E+02	1.61E+08	1.55E+02	5.39E+01	3.12E+07	2.65E+08	
3.45E+02	3.80E+02	6.20E+07	1.32E+06	1.29E+02	3.13E+07	1.62E+08	**Mean**

Table S6 Virus copy numbers after pre-treatment with ZnO-NPs at three different concentrations at 48 h post infection of cell culture with the SARS-CoV-2 Omicron-containing supernatant for each replicate (according to Figure 5 f in main text).

ZnO-NP-ref		
20 mg/ml	10 mg/ml	5 mg/ml
1.66E+02	4.66E+02	2.72E+02
2.01E+02	1.01E+03	5.95E+02

2.28E+02	5.01E+02	not detectable	
1.66E+02	4.66E+02	2.72E+02	**Mean**

Table S7 Virus Input used for cell infection after pre-treatment with ZnO-NPs for 1 h and subsequent centrifugation (according to Figure 5 a and c in main text).

VI ZnO-NP-45			VI ZnO-NP-76			VI pos Ctrl
20 mg/ml	10 mg/ml	5 mg/ml	20 mg/ml	10 mg/ml	5 mg/ml	
1.01E+02	1.15E+03	1.22E+04	6.44E+02	1.97E+03	1.86E+04	1.26E+06

Table S8 Virus Input used for cell infection after pre-treatment with ZnO-NPs for 1 h and subsequent centrifugation (according to Figure 5 e in main text).

VI ZnO-NP-ref		
20 mg/ml	10 mg/ml	5 mg/ml
1.72E+02	6.52E+02	1.07E+04
